# Diseases of the Kidneys

**Published:** 1905-01-28

**Authors:** 


(*To be continued.*)

### DISEASES OF THE KIDNEYS.

**Treatment.**—In the Oxford discussion [1] Hale White referred to the treatment of high tension in chronic renal disease by cutting off the exercise, alcohol, meat, and meat extractives taken, and any excess in drinking fluids. Constipation should also be prevented. Similarly cases with a feeble heart should have a more abundant diet, and increased but gentle exercise and even stimulants, while any tendency to obesity should be combated. Von Noorden agreed in these views, and spoke of the importance of not checking diarrhœa in uræmic states, as the bowels excrete nearly three times the nitrogen which the skin does. Sir John Moore believed that open-air treatment was of use in aiding the pulmonary elimination, and recommended benzoate of soda in 20 grain doses as often useful in uræmic coma. On the vexed question of morphine in uræmia, Tyson thought that it was safe in convulsions due to parenchymatous, but not in those due to interstitial nephritis. Broadbent pointed out that the worst symptoms of uræmia are due, not to poisons, but to the arterial tension, and that bleeding or small repeated doses of mercury have upon this a very powerful influence. In fact he regards uræmic convulsions as due to arrest of the circulation in the cortex of the brain. When the intracranial pressure is so high that the circulation is stopped by the resistance of the skull, or the heart is incapable of overcoming the resistance, stasis occurs with convulsions. Saundby mentioned instances of occasional complete recovery from chronic Bright's disease, and of its duration in other cases for 10 or 20 years while the general health remained good. Such recoveries are especially common where the disease has commenced in pregnancy, but they do occur in other types of nephritis. For chronic cases he forbids alcohol, but allows 5 oz of meat daily without meat-soups ; but if nervous symptoms arise the diet is reduced for a time even, if necessary, to milk and soda water. Persistent dropsy is a bad sign. For convulsions hot air baths and enemata of chloral, or even bleeding, are of use. There is some danger in the use of morphine, especially where the excretion of urea has been low for some time ; but in acute nephritis and that following pregnancy the risk seems very slight. J. West thinks that he has seen benefit in chronic nephritis from extracts of fresh kidneys prepared daily. The tension in granular kidney should be maintained slightly higher than normal, but reduced by nitrites or small doses of pilocarpin when necessary. Acute uræmia may be treated with inhalation of oxygen, pilocarpin, purging, and venesection. D. Newman held that surgical treatment is chiefly useful in infective nephritis, such as is caused by the bacillus coli. These cases are difficult to distinguish from non-infective inflammations, for which indeed surgical treatment is of little use. Incision of the capsule gives marked relief to renal pain, frequently stops hæmorrhage in chronic Bright's disease, gives temporary relief in anuria, dropsy, and dyspnœa, but does not give permanent cures in chronic non-infective cases. However, it is of high value for uræmia with suppression of urine in acute infective cases. The writer also recommends in place of Southey's tubes the use of a filler or tunning dish, one and a half inches across the mouth, and fitted with three feet of tubing. This is filled with boracic solution, and the mouth of the glass inverted over two small incisions in the skin, while the other end of the tube lies in a tub on the floor. The traction of the column of fluid draws serum from the tissues to a large amount, and there is no risk of the tube getting blocked as Southey's tubes do. Rose Bradford disagreed with other speakers in laying stress on the importance of the loss of albumen which may equal that contained in two pints of milk daily. In chronic disease milk diet is desirable only if the patient does not lose weight on it. The action of morphine cannot, unfortunately, be determined with certainty; roughly it seems to be useful in dyspnœa and objectionable in dropsy. Hale White also noted that if saline solutions were used for injection in uræmia they must be hypotonic, such as a ·5 per cent. chloride solution, because the serum has already a high osmotic pressure. There seems great difficulty in explaining the good effects of decapsulation. At one time it was contended that new blood-vessels entered the kidney by way of the denuded surface, but Walker Hall and Herxheimer show that in rabbits the capsule is re-formed within three weeks, and that no new vessels penetrate it. Thus there can be no relief of congestion by the formation of more numerous channels.

[1] Brit. Med. Jour., Oct. 8.

